# A 12 week longitudinal study of microbial translocation and systemic inflammation in undernourished HIV-infected Zambians initiating antiretroviral therapy

**DOI:** 10.1186/1471-2334-14-521

**Published:** 2014-09-29

**Authors:** Ashley Canipe, Takondwa Chidumayo, Meridith Blevins, Michael Bestawros, Jay Bala, Paul Kelly, Suzanne Filteau, Bryan E Shepherd, Douglas C Heimburger, John R Koethe

**Affiliations:** Division of Gastroenterology, Hepatology and Nutrition, Vanderbilt University School of Medicine, Nashville, TN USA; University Teaching Hospital, Lusaka, Zambia; Department of Biostatistics, Vanderbilt University School of Medicine, Nashville, TN USA; Division of Cardiovascular Medicine, Vanderbilt University School of Medicine, Nashville, TN USA; Vanderbilt Institute for Global Health, Nashville, TN USA; Barts & the London School of Medicine, London, UK; London School of Hygiene & Tropical Medicine, London, UK; Division of Infectious Diseases, Department of Medicine, Vanderbilt University School of Medicine, A2200-MCN, 1161 21st Avenue South, Nashville, TN USA

**Keywords:** HIV infection, Antiretroviral therapy, Microbial translocation, Nutrition, Sub-Saharan Africa, Inflammation

## Abstract

**Background:**

Undernourished, HIV-infected adults in sub-Saharan Africa have high levels of systemic inflammation, which is a risk factor for mortality and other adverse health outcomes. We hypothesized that microbial translocation, due to the deleterious effects of HIV and poor nutrition on intestinal defenses and mucosal integrity, contributes to heightened systemic inflammation in this population, and reductions in inflammation on antiretroviral therapy (ART) accompany reductions in translocation.

**Methods:**

HIV-infected, Zambian adults with a body mass index <18.5 kg/m^2^ were recruited for a pilot study to assess the relationships between microbial translocation and systemic inflammation over the first 12 weeks of ART. To assess microbial translocation we measured serum lipopolysaccharide binding protein (LBP), endotoxin core IgG and IgM, and soluble CD14, and to assess intestinal permeability we measured the urinary excretion of an oral lactulose dose normalized to urinary creatinine (Lac/Cr ratio). Linear mixed models were used to assess within-patient changes in these markers relative to serum C-reactive protein (CRP), tumor necrosis factor-α receptor 1 (TNF-α R1), and soluble CD163 over 12 weeks, in addition to relationships between variables independent of time point and adjusted for age, sex, and CD4+ count.

**Results:**

Thirty-three participants had data from recruitment and at 12 weeks: 55% were male, median age was 36 years, and median baseline CD4+ count was 224 cells/μl. Over the first 12 weeks of ART, there were significant decreases in serum levels of LBP (median change -8.7 μg/ml, p = 0.01), TNF-α receptor 1 (-0.31 ng/ml, p < 0.01), and CRP (-3.5 mg/l, p = 0.02). The change in soluble CD14 level over 12 weeks was positively associated with the change in CRP (p < 0.01) and soluble CD163 (p < 0.01). Pooling data at baseline and 12 weeks, serum LBP was positively associated with CRP (p = 0.01), while endotoxin core IgM was inversely associated with CRP (p = 0.01) and TNF-α receptor 1 (p = 0.04). The Lac/Cr ratio was not associated with any serum biomarkers.

**Conclusions:**

In undernourished HIV-infected adults in Zambia, biomarkers of increased microbial translocation are associated with high levels of systemic inflammation before and after initiation of ART, suggesting that impaired gut immune defenses contribute to innate immune activation in this population.

**Electronic supplementary material:**

The online version of this article (doi:10.1186/1471-2334-14-521) contains supplementary material, which is available to authorized users.

## Background

The epidemics of human immunodeficiency virus (HIV), chronic undernutrition, and food insecurity disproportionately affect Sub-Saharan Africa, and nutritional deficits contribute to increased morbidity and mortality among HIV-infected individuals in the region [1]. Undernourished (defined as body mass index [BMI] < 18.5 kg/m^2^) HIV-infected adults have a strikingly high early mortality rate after initiation of antiretroviral therapy (ART), over 25% in the first 90–180 days in some studies, which is 4–6 fold higher than HIV-infected patients who are adequately nourished and is independent of the degree of immunosuppression (CD4+ T-cell count) [2–5]. The mechanism underlying this high early mortality is unknown and likely multifactorial, but prior studies have found heightened systemic inflammation to be a major risk factor [6, 7].

In studies of predominantly well-nourished individuals, microbial translocation has been proposed as an etiology of the persistent immune activation observed in patients with both untreated and treated HIV. However, there are few reports on the contribution of microbial translocation to the exceptionally high levels of systemic inflammation observed in patients with comorbid malnutrition and advanced HIV disease in resource-limited settings [8–10].

HIV infection depletes CD4+ cells in the gastrointestinal mucosa, particularly Th17 regulatory T-cells integral to defense against bacterial, fungal, and parasitic pathogens, and impairs tight junctions between epithelial cells, resulting in altered intestinal integrity and increased translocation of microbes from the intestinal lumen to the circulation [11–15]. Increased microbial translocation, as measured by circulating lipopolysaccharide (LPS; a component of the bacterial cell wall), anti-endotoxin antibodies, soluble CD14 (sCD14), and other biomarkers is associated with accelerated HIV disease progression and a higher risk of mortality [16, 17]. Irrespective of HIV infection status, chronic undernutrition also impairs intestinal mucosal integrity and permits increased microbial translocation in a manner similar to advanced HIV infection, suggesting that the presence of both conditions in the same host may have compounding effects [18, 19].

We hypothesized that the high levels of systemic inflammation observed in undernourished adults with advanced HIV in sub-Saharan Africa are due in part to excessive microbial translocation, and reductions in microbial translocation following treatment initiation accompany a normalization of this inflammatory response. To test this hypothesis, we conducted a pilot study to measure intestinal permeability, microbial translocation, and systemic inflammation among undernourished, HIV-infected African adults starting ART.

## Methods

This study was nested within a prospective, randomized-controlled trial of nutritional support to reduce early ART mortality (the Nutritional Support for African Adults Starting Antiretroviral Therapy [NUSTART] study – trial registration #PACTR201106000300631). Briefly, the NUSTART study was a randomized controlled trial of a lipid-based nutritional supplement, with or without additional vitamins and minerals, conducted in Zambia and Tanzania between 2011 and 2013 among HIV-infected, ART-eligible adults with clinical malnutrition (defined as a BMI < 18.5 kg/m^2^) [20]. We enrolled a subset of trial participants from Zambia into a prospective cohort to evaluate markers of intestinal permeability, microbial translocation, and systemic inflammation from recruitment through the first 12 weeks of ART (inclusive of nutritional supplementation from enrollment to 6 weeks after starting ART).

Potentially eligible participants were evaluated by study staff at local health clinics and referred to University Teaching Hospital in Lusaka, Zambia for enrollment in the parent NUSTART trial and all study procedures. Inclusion criteria were at least 18 years of age, ART-naïve (except for prior receipt of Prevention of Mother To Child Transmission regimens), ART-eligibility as determined by CD4+ T-cell count <350 cells/μl, BMI < 18.5 kg/m^2^, willingness to undertake intensive ART follow-up in the study clinic, and not pregnant.

Enrollment for this sub-study occurred from August 2012 through December 2012. Prior to starting ART and after 12 weeks of treatment, microbial translocation across the gut wall was assessed by measuring serum levels of LPS binding protein (LBP), endotoxin core IgG (EndoCAb IgG) and EndoCAb IgM (two antibodies previously shown to rise and fall, respectively, in response to circulating LPS levels) [21], and sCD14 using ELISA (Hycult Biotech, Uden, Netherlands). To assess systemic inflammation we measured serum levels of C-reactive protein (CRP), soluble TNF-α receptor 1, and soluble CD163 (sCD163, a marker of monocyte activation) using ELISA (R&D Systems, Minneapolis, MN USA). Intestinal permeability was measured using the fractional absorption and urinary excretion of lactulose method (Lac/Cr ratio). Patients ingested a 10 g dose of lactulose powder (Kristalose, Cumberland Pharmaceuticals, Nashville, TN, USA) dissolved in water, and urine was collected over a 5 hour period. The urine concentration of lactulose in mmol/l was measured using an enzymatic, spectrophotometric technique and normalized to urine creatinine in mol/l (Oxford Biomedical Research, Rochester Hills, MI, USA) [22, 23]. Participants who reported more than four bowel movements in the 24 hours preceding the enrollment were excluded from the lactulose urinary excretion testing because lactulose is a cathartic agent which could exacerbate diarrhea and contribute to volume depletion.

Participant clinical and demographic characteristics were expressed as percentages or as median values with interquartile range. The change in each biomarker from recruitment to 12 weeks of ART was assessed using the Wilcoxon signed rank test. Local polynomial regression fitting (loess) curves were fit to provide smoothed trends. We assessed the relationships between permeability, microbial translocation and inflammation biomarkers irrespective of time point (baseline or 12 weeks) using a linear mixed model with a random effect for the patient to account for correlation that may arise from measurements taken from the same patient, and adjusted for age, sex, and pre-treatment CD4+ T-cell count. The permeability and translocation biomarkers (LBP, EndoCAb IgG and IgM, sCD14, and urinary Lac/Cr ratio) were log-transformed whereas inflammation biomarkers (CRP, TNF-α R1, and sCD163) remained on the unit scale. The primary analysis included the 33 participants evaluated at both recruitment and 12 weeks; a secondary analysis incorporated the recruitment visit data from 27 additional patients without 12 week data. Lastly, we assessed the relationship between the change in permeability and translocation biomarkers with the change in inflammation over the 12 weeks (i.e., within-patient comparison) using ordinary least squares regression, with adjustment for the baseline biomarker values so that the effect measure is independent of baseline.

Analyses were performed using R (version 2.12.1; http://www.r-project.org). Analysis scripts are posted at http://biostat.mc.vanderbilt.edu/wiki/Main/ArchivedAnalyses. The study was approved by the Institutional Review Board at Vanderbilt University and the Biomedical Research Ethics Committee of the University Teaching Hospital in Lusaka, Zambia, and all patients provided written informed consent.

## Results

Sixty ART-naïve adults were recruited for this study between August and December 2012, and 33 returned at 12 weeks for the clinical assessment and laboratory studies. Of the 27 patients without week 12 data, one-third (n = 9) had never started ART, 2 died, 3 withdrew due to relocation, pregnancy, or acute illness, and 13 declined to return for week-12 measurements. Those included and excluded from the analysis did not differ according to age, sex, or pre-treatment BMI, serum hemoglobin, or CD4+ T-cell count (p > 0.05 for all). Among the 33 patients included in the follow-up analysis, 55% were male and at enrollment, the median age was 36 years (interquartile range [IQR] 31–42), and the median CD4+ T-cell count was 224 cells/μl (IQR 151–256); all participants were undernourished according to WHO criteria (i.e. BMI <18.5 kg/m^2^), with a median BMI of 16.7 kg/m^2^ (IQR 15.4-17.6). Table 1 shows the clinical characteristics of the 33 included cohort patients. Ten of the participants had fewer than 4 pairs of laboratory data and could not be included in some of the statistical models. Compared to the other 23 patients, those with fewer than 4 data pairs were similar by age, sex, hemoglobin or CD4+ T-cell count (p > 0.05 for all), but had lower pre-treatment BMI (15.6 versus 17.4 kg/m^2^; p = 0.02).

**Table 1:** **Description of the cohort (n = 33)**

Demographic and clinical characteristics		
Female sex, n (%)	15 (45%)	
Age (median years, IQR)	36 (31–42)	
Baseline BMI (median kg/m^2^, IQR)	16.7 (15.4 - 17.6)	
Week 12 BMI (median kg/m^2^, IQR)	17.6 (16.5 - 19.0)*	
Baseline CD4+ count (median cells/μL, IQR)	224 (151–256)	
Week 12 CD4+ count (median cells/μL, IQR)	349 (275–426)*	
Median serum and urine biomarker values pre-treatment and after 12 weeks of antiretroviral therapy, median (IQR)		
**Biomarker**	**Recruitment**	**Week 12 of ART**
Lipopolysaccharide binding protein (μg/ml)	46.7 (33.6 – 74.3)	38.4 (31.1 – 44.4)*
Soluble CD14 (μg/ml)	2.4 (2.0 - 2.6)	2.5 (2.0 - 3.1)
Endotoxin core IgM (units/ml)	14.1 (12.7 - 20.0)	17.7 (11.7 - 22.7)
Endotoxin core IgG (units/ml)	38.4 (23.9 - 86.6)	32.9 (16.1 - 94.5)
C-reactive protein (mg/l)	18.3 (4.5 - 64.8)	6.4 (3.8 - 19.7)*
TNF-α receptor 1 (ng/ml)	1.3 (0.8 – 2.4)	1.0 (0.6 – 1.3)*
Soluble CD163 (ng/ml)	684 (511–1022)	531 (376–1040)
Urine lactulose-to-creatinine ratio (mmol/mol)	29.3 (12.6 – 55.2)	34.0 (14.1 – 86.3)

*Abbreviation: ART* antiretroviral therapy, *BMI* body mass index, *TNF-α* tumor necrosis factor-α, *IQR* interquartile range.

*p < 0.05 for change at 12 weeks.

Participants reported a range of clinical complaints at enrollment including cough (66%), subjective fever (41%), pain with swallowing (13%), abdominal pain (44%), diarrhea (28%), shortness of breath (25%), and vomiting (9%), but there were no significant differences in any of the baseline microbial translocation or inflammation biomarker levels between participants with and without these complaints (data not shown). Similarly, there were no significant differences in biomarker levels between those on therapy for tuberculosis (22%) or with recent diarrheal illness (4%) at enrollment.

**Figure 1:**
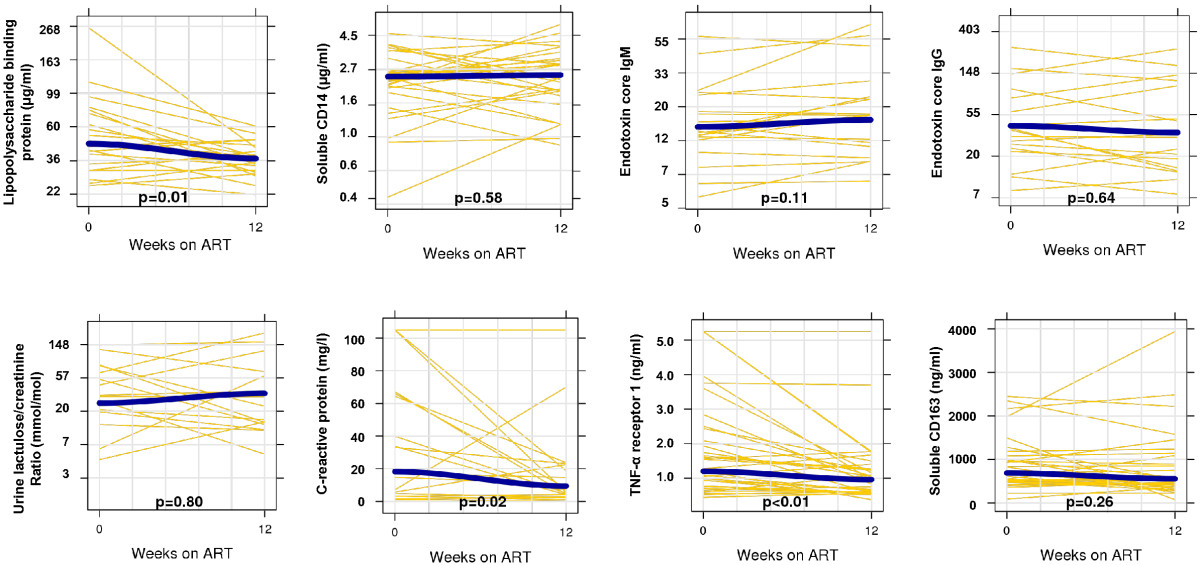
**Changes in microbial translocation and inflammation biomarkers after 12 weeks of antiretroviral therapy.** Yellow lines represent paired samples and the blue lines represent loess curves. As in the statistical models, the permeability and translocation biomarkers are log-transformed (with the y-axis labeled in the original scale) and the inflammation biomarkers are on the unit scale. Wilcoxon signed rank test assessed statistical significance.

From recruitment to 12 weeks of ART there was a significant decrease in serum levels of LBP (median change -8.7 μg/ml, p = 0.01), TNF-α receptor 1 (-0.31 ng/ml, p < 0.01), and CRP (-3.5 mg/l, p = 0.02). An increase in EndoCAb IgM antibody (0.6 units/ml, p = 0.11) did not reach statistical significance. The Figure 1 shows the changes in the biomarkers from recruitment to 12 weeks of ART among all patients with paired samples, with smoothed curves fit to demonstrate overall trends, while the Additional file 1: Figure S1 shows the changes for inflammatory biomarkers as a function of changes in microbial translocation biomarkers.

Associations between pooled baseline and 12-week biomarkers of intestinal permeability, microbial translocation and inflammation were assessed using linear mixed models adjusted for age, sex, and baseline CD4+ T-cell count (Table 2). Serum LBP level was positively associated with CRP (p = 0.01), and closely related to TNF-α receptor 1 and sCD163 (p = 0.08 and p = 0.13, respectively). Serum EndoCAb IgM level (a circulating antibody shown in prior studies to rise in response to decreasing microbial translocation) was inversely associated with serum levels of CRP (p = 0.01) and TNF-α receptor 1 (p = 0.04) [21]. Serum sCD14 was associated with CRP (p < 0.01) and sCD163 (p < 0.01), and closely related to TNF-α receptor 1 (p = 0.07). We did not detect statistically significant relationships between EndoCAb IgG or the urine Lac/Cr ratio and the inflammatory biomarkers. Results were similar when recruitment data from participants without a week 12 visit were included in the analysis.

**Table 2:** **Regression coefficients estimating the associations between biomarkers of microbial translocation and inflammation**

	Inflammation biomarkers		
**Microbial translocation biomarkers**	C-reactive protein	Soluble CD163	TNF-α receptor 1
	Estimate (95% CI)	Estimate (95% CI)	Estimate (95% CI)
	p-value; model size	p-value; model size	p-value; model size
Lipopolysaccharide binding protein	**28.9 (8.6, 49.1)**	169 (−14, 352)	773 (−11, 1559)
	**p = 0.01;** n = 40	p = 0.13; n = 40	p = 0.08; n = 40
Endotoxin core IgM antibody	**−25.7 (−42.7, −8.7)**	−4.4 (−164, 155)	**−713 (−1439, 12)**
	**p = 0.01;** n = 38	p = 0.90; n = 38	**p = 0.04;** n = 40
Endotoxin core IgG antibody	−4.9 (−17.1, 7.4)	39 (−58, 137)	−324 (−762, 113)
	p = 0.58; n = 36	p = 0.76; n = 36	p = 0.54; n = 38
Soluble CD14	**44.7 (21.3, 68.2)**	**595 (289, 902)**	620 (8, 1232)
	**p < 0.01;** n = 40	**p < 0.01;** n = 62	p = 0.07; n = 62
Urine lactulose/creatinine ratio (linear)	0.22 (−0.02, 0.45)	11 (−118, 140)	141 (−186, 468)
	p = 0.33; n = 27	p = 0.68; n = 38	p = 0.81; n = 39

Results from linear mixed models pooling data across both recruitment and 12 weeks of ART. Models adjusted for age, sex, and baseline CD4+ T-cell count.

Microbial translocation biomarkers (regression predictor) are log-transformed and inflammation biomarkers (regression outcome) remain on the unit scale.

Model size represents total observations (recruitment and 12 week). Bold values represent statistically significant findings.

In the linear models, the change in sCD14 from recruitment to 12 weeks was associated with the change in CRP (p = 0.02) and sCD163 (p = 0.01) (Table 3). However, the change in EndoCAb IgM at 12 weeks was also positively associated with the change in sCD163 (p = 0.02), which was unexpected given the results from the pooled analyses. Changes in other translocation biomarkers over 12 weeks were not significantly associated with changes in inflammation.

**Table 3:** **Paired changes in microbial translocation and inflammation biomarkers over 12 weeks of ART**

	Inflammation biomarkers (12 week change)		
**Microbial biomarkers (12 week change)**	C-reactive protein	Soluble CD163	TNF-α receptor 1
	Estimate (95% CI)	Estimate (95% CI)	Estimate (95% CI)
	p-value; model size	p-value; model size	p-value; model size
Lipopolysaccharide binding protein	−19 (−72, 33)	13 (−510, 536)	757 (−1256, 2769)
	p = 0.45; n = 20	p = 0.96; n = 20	p = 0.44; n = 20
Endotoxin core IgM antibody	−18 (−61, 25)	**436 (72, 800)**	−74 (−1628, 1480)
	p = 0.38; n = 19	**p = 0.02**; n = 19	p = 0.92; n = 30
Endotoxin core IgG antibody	−1.2 (−34, 31)	168 (−86, 423)	−500 (−1413, 412)
	p = 0.94; n = 17	p = 0.18; n = 17	p = 0.26; n = 18
Soluble CD14	**38 (7, 69)**	**697 (183, 1212)**	553 (−208, 1313)
	**p = 0.02**; n = 20	**p = 0.01**; n = 30	p = 0.15; n = 20
Urine lactulose/creatinine ratio (linear)	−0.27 (−22, 21)	154 (−79, 388)	121 (−86, 329)
	p = 0.97; n = 11	p = 0.17; n = 14	p = 0.22; n = 14

Linear models estimate the relationships between 12-week changes in the log-transformed microbial translocation biomarkers (regression predictor) and 12-week changes in the inflammatory biomarkers (regression outcome) using ordinary least squares regression, adjusted for baseline biomarker values. Model size represents number of paired observations. Bold values represent statistically significant findings.

Lastly, we assessed the relationship between the urinary Lac/Cr ratio, our biomarker of intestinal permeability, and the microbial translocation and inflammation biomarkers. We failed to detect any association of Lac/Cr ratio with serum levels of LBP, EndoCAb IgM, EndoCAb IgG, or sCD14 in the linear mixed models, nor was the change in Lac/Cr over 12 weeks associated with the change in any of the translocation biomarkers over the same period. Similarly, the Lac/Cr ratio was not associated with the measures of systemic inflammation.

## Discussion

In this pilot study of undernourished adults starting ART in sub-Saharan Africa, serum levels of circulating CRP and other inflammatory biomarkers were markedly elevated prior to treatment initiation and after 12 weeks, and there was evidence that reductions in microbial translocation biomarkers, particularly LBP, accompanied reductions in inflammatory biomarkers. However, while we observed close associations between several pairs of translocation and inflammation biomarkers, there was marked heterogeneity both between and within participants. This suggests the hypothesis that microbial translocation is a primary driver of inflammation in HIV-infected adults does not adequately capture what is likely a complex interplay of factors promoting innate immune activation in malnourished adults with advanced HIV disease.

Microbial translocation in undernourished, HIV-infected adults is likely due to the combined effects of HIV enteropathy, environmental enteropathy, and malnutrition on the intestinal mucosal barrier. HIV enteropathy generally occurs in advanced disease (e.g., CD4+ T-cell count <200 cells/μl) and is characterized by changes in villous morphology, absorption, and mucosal permeability [24, 25]. Mucosal T-cell depletion, particularly loss of the Th17 cells important for bacterial, fungal, and parasitic defense, in conjunction with impaired cellular tight junctions has been proposed as an underlying mechanism [13, 14, 26]. Environmental enteropathy is common in many tropical regions, including Zambia, and is morphologically similar to HIV enteropathy [27]. It is thought to result from a combination of recurrent, transient infections with pathogenic bacteria and altered intestinal microbiota resulting in chronic T-cell mediated enteric inflammation, malabsorption, impairment of mucosal integrity, and reduced expression of antimicrobial peptides [27–30]. Lastly, malnutrition is associated with hypoalbuminemia and bowel wall edema, impaired adaptive immune responses, and reduced mucosal integrity [18, 19, 31].

Recent studies suggest that interventions to alter intestinal microbiota and mucosal cellular function in comorbid malnutrition and advanced HIV disease could provide clinical benefits for these patients, though much of this research remains at an early stage. There is evidence to suggest that probiotics can alter gastrointestinal tract microbiota, with beneficial effects on mucosal immunity [32]. In the HIV-infected population, administration of probiotics and other specialized nutritional products has led to modest improvements of CD4+ T-cell counts, reduced CD4+ T cell activation, and fewer gastrointestinal disorders [33–36]. In the mouse model, glucagon-like peptide and probiotics improved intestinal integrity and reduced inflammation in obese mice [37]. Other promising non-human trials have demonstrated that vaccines may improve gastrointestinal tract immunity by recruiting IgA secreting plasma cells to the mucosa or decreasing the local HIV burden [38, 39], and the use of a bovine serum immunoglobulin in adults with HIV enteropathy improved duodenal absorptive function and increased mucosal CD4+ T cell density [40].

While increased microbial translocation and systemic inflammation are both associated with poorer health outcomes in HIV infection, further research is needed to understand the clinical consequences in undernourished African patients and to determine if prior findings in US and European cohorts are applicable to this population. In a US cohort of ART-treated adults, higher serum levels of intestinal fatty acid binding protein and lower zonulin-1, both indicative of increased gut permeability, were associated with increased all-cause mortality [41]. In Italian HIV patients, serum LPS levels predicted disease progression independently of age, CD4+ T-cell count, viral load, or duration of infection, and higher circulating LPS levels after ART initiation were associated with greater CD4+ and CD8+ T-cell activation and poor CD4+ T-cell recovery [16, 42]. Increased microbial translocation is also associated with dyslipidemia, insulin resistance, and increased risk of cardiovascular events in both HIV-infected and uninfected individuals [43–45], and persistently elevated CRP and other inflammation biomarkers predispose to fatal and non-fatal cardiovascular events [46, 47]. However, conclusions regarding long-term health outcomes in African populations based on these studies are not warranted given differences in populations, background disease risk, methodology, and other factors. Further epidemiologic studies are needed to determine how very high levels of both microbial translocation and inflammation affect risk for cardiovascular, metabolic and other non-communicable diseases among HIV patients in sub-Saharan Africa.

While sCD14 has been posited as a marker of microbial translocation in prior studies of HIV-infected and uninfected populations, the association between sCD14 and inflammatory markers observed in our study and prior studies should be interpreted with caution [8, 9, 16, 48–50]. A major uncertainty is whether high circulating sCD14 levels observed in HIV patients derive from intestinal or hepatic immune cells (e.g., intestinal monocytes or Kupffer cells exposed to LPS via the portal vein) and thus reflect increased intestinal microbial transit, or whether the increased sCD14 derives from immune cells in more distant tissues and should be classified as a general biomarker of systemic inflammation [51]. This uncertainty reduces the utility of sCD14 as an indicator of microbial translocation.

Our measurements of Lac/Cr ratio were not associated with any of our serum markers of microbial translocation, suggesting that intestinal permeability as measured by this method may not represent the same epithelial defects involved in increased microbial transit. In our cohort, the median baseline Lac/Cr ratio value of 29.3 mmol/mol was approximately 3-fold higher than in healthy controls in a prior study of European adults, but considerably lower than patients with active Crohn’s disease [52]. Furthermore, we did not observe a clinically significant change in the Lac/Cr ratio over 12 weeks, which may indicate that while defenses against microbial translocation improved on ART, paracellular absorption from the gut lumen remained elevated. However, the use of the Lac/Cr ratio in HIV-infected, malnourished adults requires further validation given the chronic dehydration, potential HIV- and nutrition-related renal dysfunction, and reduced muscle mass present in this population.

Our pilot study was limited by small sample size and incomplete follow-up of some participants. The lack of both a non-malnourished, HIV-infected control group and a malnourished, uninfected control group made it impossible to explore the independent, and potentially synergistic, contributions of HIV and malnutrition to microbial translocation. The inverse association we observed between EndoCAb IgM and inflammation markers was expected based on prior sepsis studies showing that EndoCAb IgM levels vary inversely with serum endotoxin in the post-acute phase; however, we did not see any association between EndoCAb IgG and the outcomes of interest [21]. One possibility is that the EndoCAb IgG required adjustment for total IgG level, which is both high and variable in HIV infection. Future studies would also benefit from quantification of serum LPS or bacterial DNA and analysis of intestinal histology, rather than the surrogate markers of microbial translocation we used. Lastly, our study did not measure plasma HIV-1 RNA levels, which may have had independent pro-inflammatory effects and confounded our analysis, particularly if not all participants reached viral suppression after 12 weeks of treatment.

## Conclusions

Our findings suggest that excessive microbial translocation across the bowel wall contributes to the high levels of systemic inflammation observed in undernourished adults with advanced HIV disease in sub-Saharan Africa. A high degree of translocation is likely due to the compounding effects of HIV enteropathy, environmental enteropathy, and malnutrition on mucosal barrier integrity and local immune defenses. Given the known adverse effects of heightened inflammation and microbial translocation on morbidity, mortality, and immune recovery after ART initiation, the development of interventions to improve gastrointestinal health in these patients may have important benefits for clinical outcomes.

## Electronic supplementary material

Additional file 1: Figure S1: Directional change in inflammation and microbial translocation biomarkers over 12 weeks among individual participants. Legend: Arrows represent the directional change in pairs of inflammation and microbial translocation biomarkers for each participant over the 12 weeks of follow-up. (PPT 2 MB)

Below are the links to the authors’ original submitted files for images.

Authors’ original file for figure 1
