# Supplementary material for: A 12 week longitudinal study of microbial translocation and systemic inflammation in undernourished HIV-infected Zambians initiating antiretroviral therapy
Source: BMC Infect Dis. 2014 Sep 29;14:521. doi: 10.1186/1471-2334-14-521 (PMC4261887; doi:10.1186/1471-2334-14-521)
Supplement: Supplementary file 1 — Additional file 1: Figure S1: Directional change in inflammation and microbial translocation biomarkers over 12 weeks among individual participants. Legend: Arrows represent the directional change in pairs of inflammation and microbial translocation biomarkers for each participant over the 12 weeks of follow-up. (PPT 2 MB) [file 12879_2014_3842_MOESM1_ESM.ppt]

## Slide 1
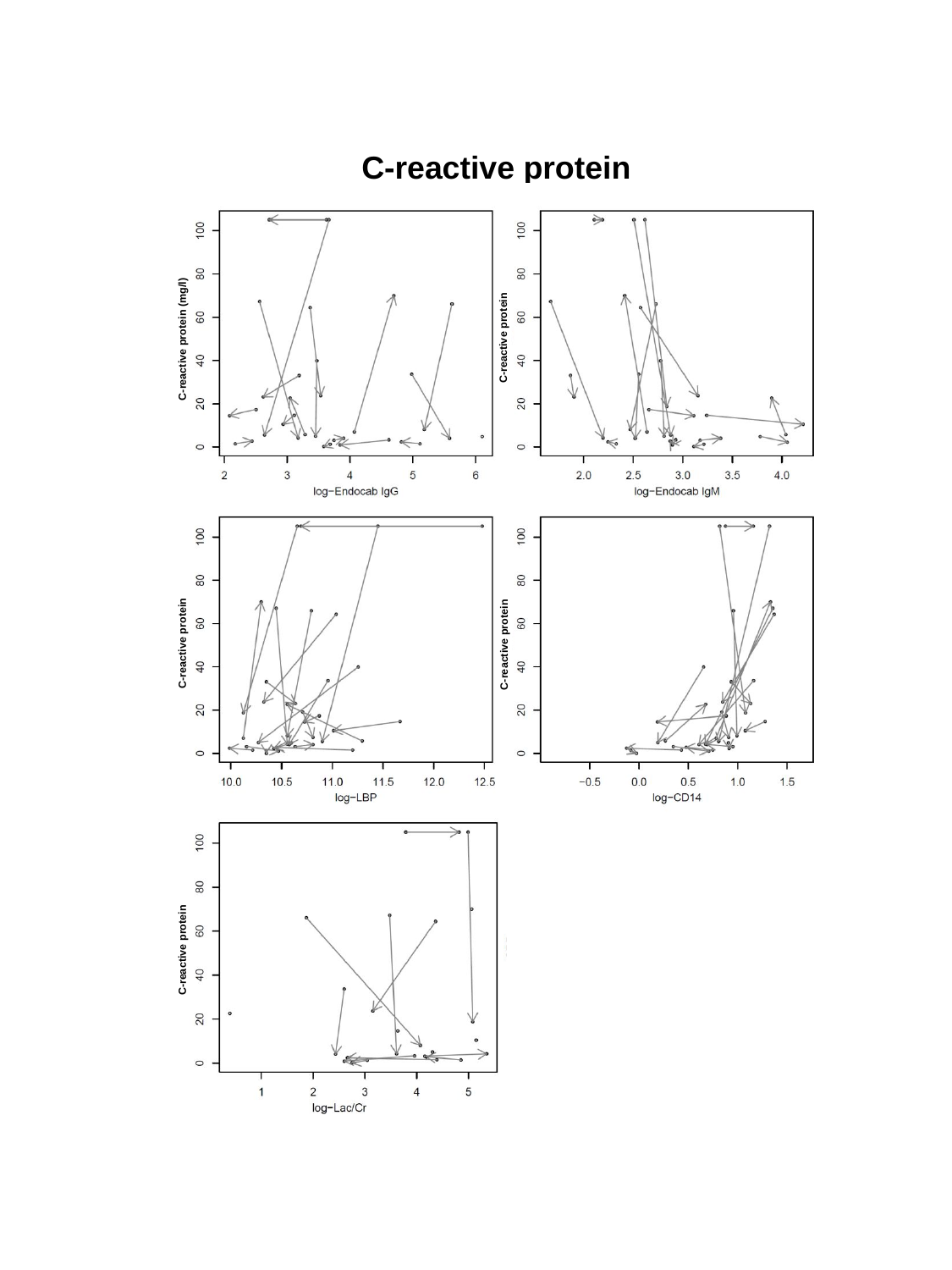

C-reactive protein
C-reactive protein
C-reactive protein (mg/l)
C-reactive protein
C-reactive protein
C-reactive protein

## Slide 2
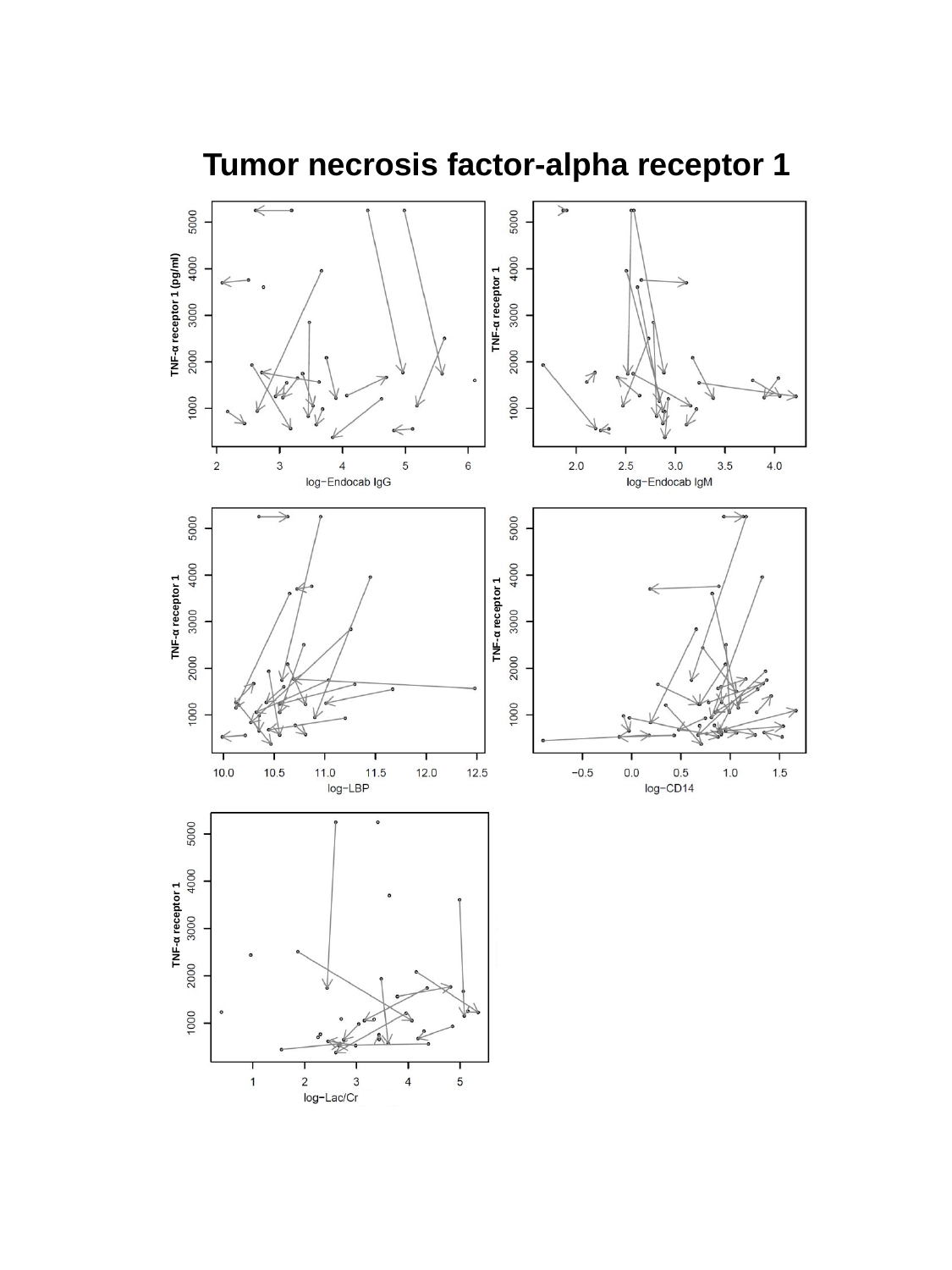

Tumor necrosis factor-alpha receptor 1
TNF-α receptor 1
TNF-α receptor 1 (pg/ml)
TNF-α receptor 1
TNF-α receptor 1
TNF-α receptor 1

## Slide 3
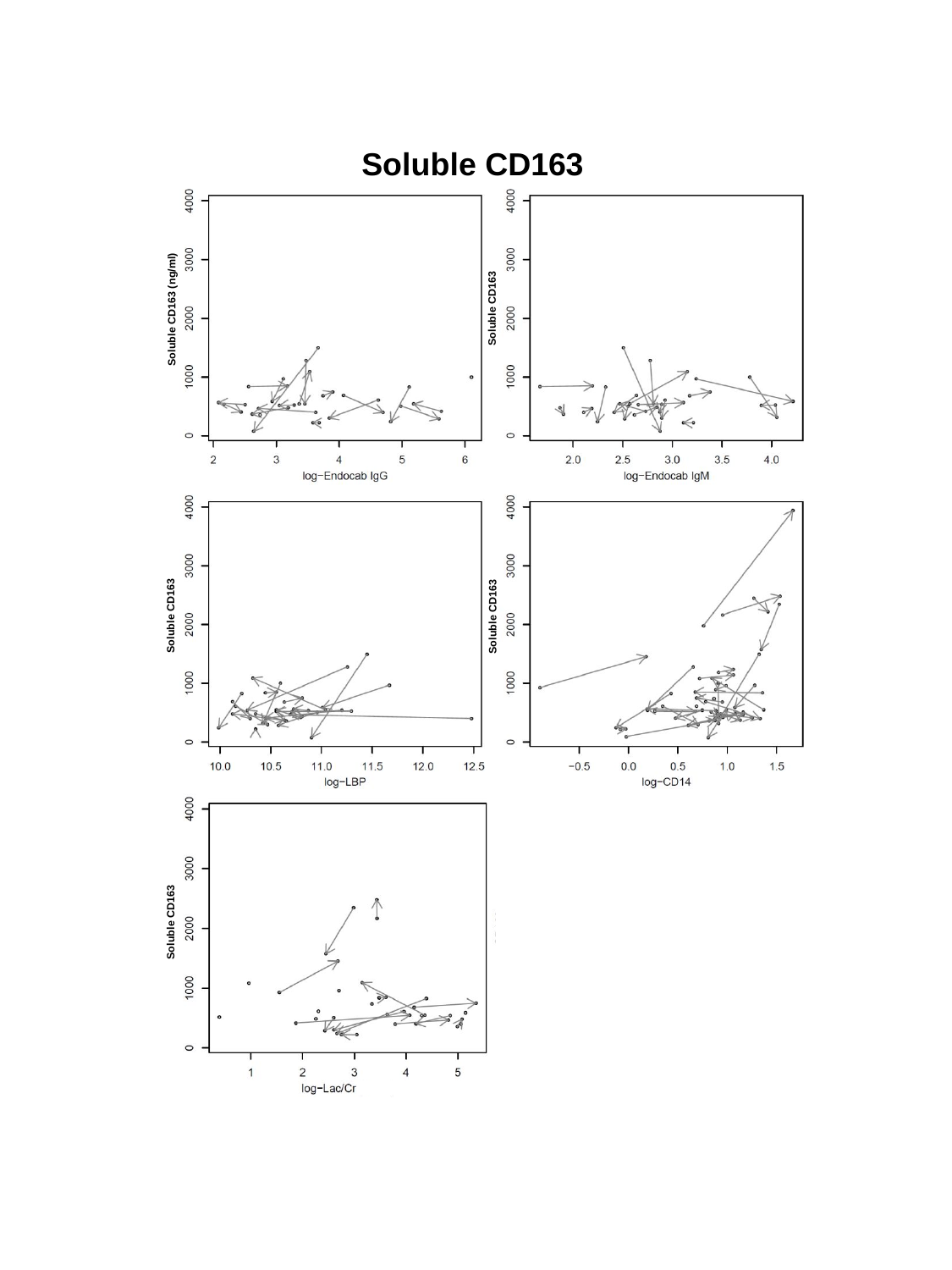

Soluble CD163
Soluble CD163
Soluble CD163 (ng/ml)
Soluble CD163
Soluble CD163
Soluble CD163
