# Supplementary figures and images for: A 12 week longitudinal study of microbial translocation and systemic inflammation in undernourished HIV-infected Zambians initiating antiretroviral therapy
Source: BMC Infect Dis. 2014 Sep 29;14:521. doi: 10.1186/1471-2334-14-521 (PMC4261887; doi:10.1186/1471-2334-14-521)

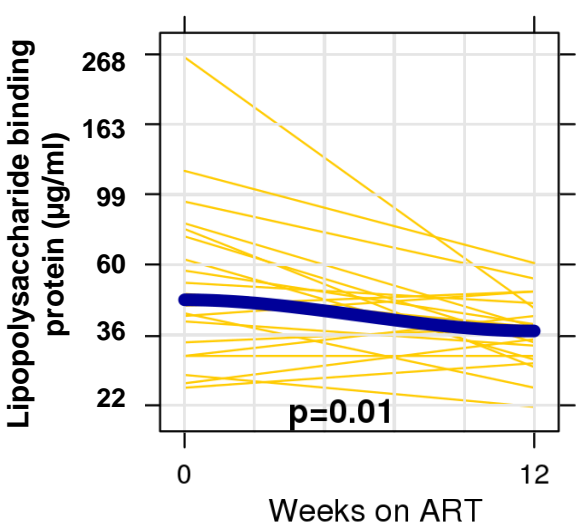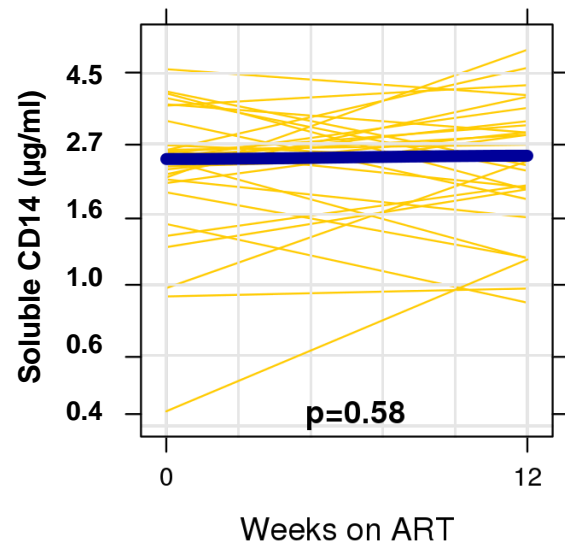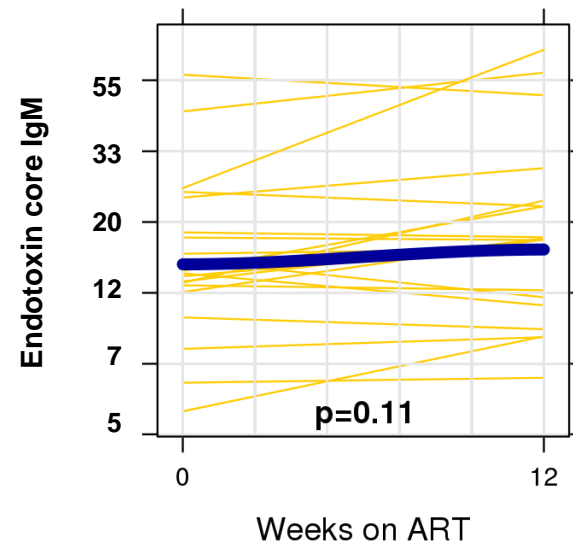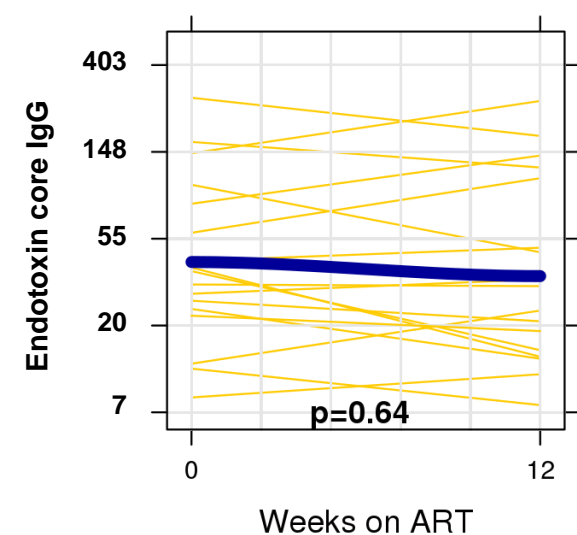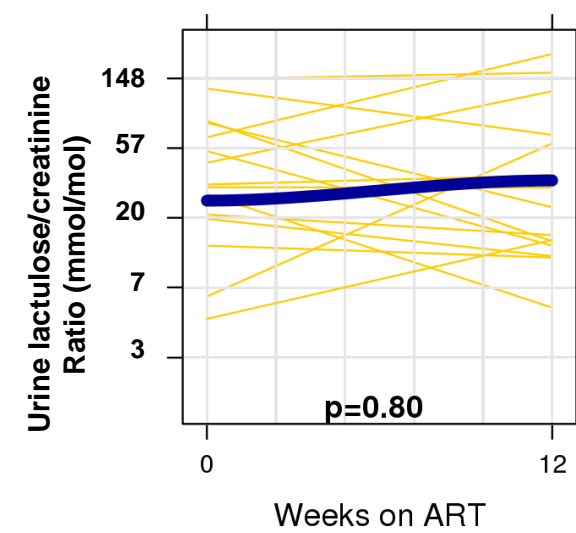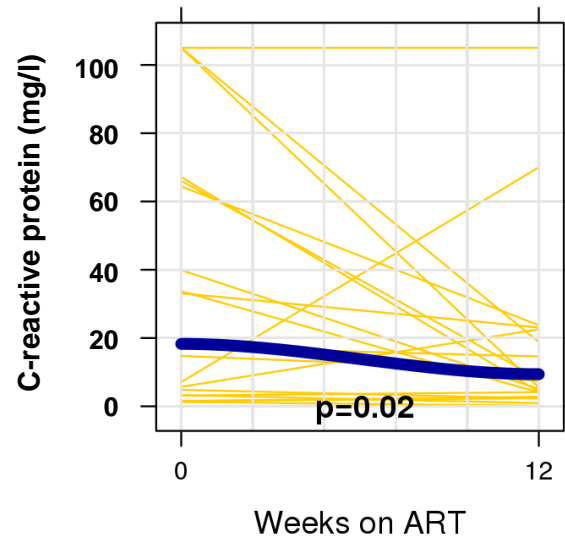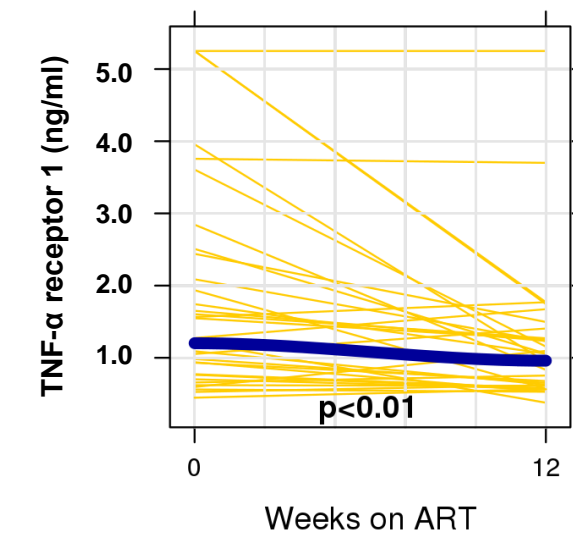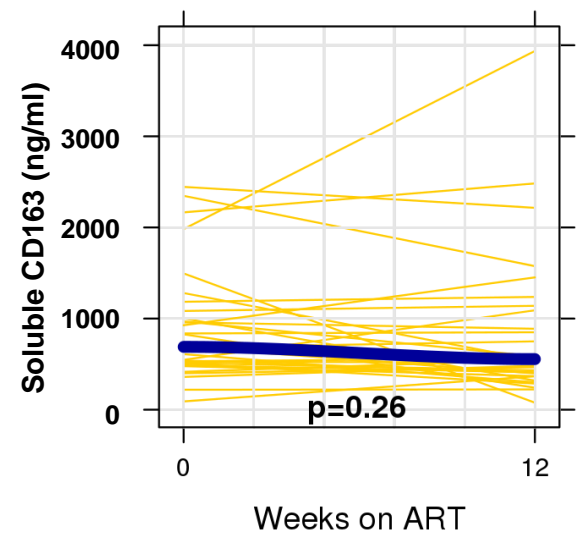

Supplement: Supplementary file 2 — Authors’ original file for figure 1 [file 12879_2014_3842_MOESM2_ESM.pdf]
